# Supplementary material for: Neuronal and Astrocytic Differentiation from Sanfilippo C Syndrome iPSCs for Disease Modeling and Drug Development
Source: J Clin Med. 2020 Feb 28;9(3):644. doi: 10.3390/jcm9030644 (PMC7141323; doi:10.3390/jcm9030644)
Supplement: Supplementary file 1 [file jcm-09-00644-s001.pdf]

Supplementary

Table S1. Products used for human iPSCs maintenance.

Product	Reference	Company	Application
mTeSR™ Plus	#05825	STEMCELL Technologies	iPSC medium
Penicillin Streptomycin (P/S)	#15140122	Thermo Fisher Scientific	Cell medium
Matrigel	#354234	Corning	Matrix
StemPro Accutase Cell Dissociation Reagent (Accutase)	#A1110501	Thermo Fisher Scientific	Cell passage
Thiazovivin (TZV)	#72252	STEMCELL Technologies	iPSC survival
ROCK inhibitor (RI)	#Y-27632	STEMCELL Technologies	iPSC survival

Table S2. Products used for lentiviral production.

Product	Reference	Company	Application
M2-rtTA (rtTA)	#20342	Addgene	Viral production
pTet-O-Ngn2-puro (Ngn2)	#52047	Addgene	Viral production
pMD2.G	#12259	Addgene	Viral production
pRSV-Rev	#12253	Addgene	Viral production
pMDLg/pRRE	#12251	Addgene	Viral production
tetO-Sox9-Puro (Sox9)		Henrik Ahlenius group	Viral production
tetO-Nfib-Hygro (Nfib)		Henrik Ahlenius group	Viral production
DMEM	#D5796	Sigma	Virus resuspension

Table S3. Products used for the generation of induced neurons and astrocytes from iPSCs.

Product	Reference	Company	Application
Doxycycline (Dox)	#10592-13-9	Thermo Fisher Scientific	tetO induction
High Pure RNA Isolation Kit	#11828665001	Roche	RNA extraction
Cell scrapers	#179693	LabClinics	Neural passage
PFA 4%	#30525-89-4	VWR	Cell fixation
DMEM/F12	#11330057	Thermo Fisher Scientific	Neural and astrocyte medium
Neurobasal	#21103049	Thermo Fisher Scientific	Neural and astrocyte medium
B-27 supplement	#17504044	Thermo Fisher Scientific	Neural and astrocyte medium
N2 supplement	#17502048	Thermo Fisher Scientific	Neural and astrocyte medium
GlutaMAX	#35050061	Thermo Fisher Scientific	Neural and astrocyte medium
Puromycine	#A1113803	Thermo Fisher Scientific	Cell selection
Sterile filters of 40 μm porus	#352340	Corning	Neural passage
FBS	#16000044	Thermo Fisher Scientific	Astrocyte medium
NEAA	#11140035	Thermo Fisher Scientific	Astrocyte medium
bFGF	#100-18B	Peprotech	Astrocyte medium
CNTF	#450-13	Peprotech	Astrocyte medium
BMP4	#120-05ET	Peprotech	Astrocyte medium

Table S4. Products used for siRNA transfection, immunocytochemistry, RT-qPCR, ELISA and protein quantification.

Product	Reference	Company	Application
Lipofectamine RNAiMAX	#13778075	Thermo Fisher Scientific	siRNA transfection
siRNA Silencer® Select (siRNA)	#4392420	Thermo Fisher Scientific	siRNA transfection
siRNA *EXTL2* gene	AssayID: si4899		
Negative Control siRNA	#4459405	Thermo Fisher Scientific	siRNA transfection
Triton-X 100	#28817.295	VWR	Immunocytochemistry
Normal Donkey Serum	#S30-100M	Merck Millipore	Immunocytochemistry
DAPI	#D1306	Thermo Fisher Scientific	Immunocytochemistry
Hoechst 33342	#H3570	Thermo Fisher Scientific	Immunocytochemistry
MOWIOL	#475904	Merck Millipore	Immunocytochemistry
High Capacity cDNA Reverse Transcription kit	#4368814	Applied Biosystems	RT-qPCR
RNase Inhibitor	#N8080119	Applied Biosystems	RT-qPCR
LightCycler 480 Probes Master	#04887301001	Roche	RT-qPCR
DC™ Protein Assay	#5000111	Bio-Rad	Protein quantification

Table S5. Antibodies used for immunocytochemistry.

Product	Reference	Dilution	Company
rabbit anti-TUJ1 primary antibody	#MMS-435P	1:500	Covance
Mouse anti-LAMP2 primary antibody	#H4B4-c	1:100	Hybridoma Bank
chicken anti-MAP2 primary antibody	#ab5392	1:5000	Abcam
mouse anti-VIM primary antibody	# M0725	1:250	Dako
rabbit anti-S100B primary antibody	#287003	1:400	Synaptic systems
donkey anti-mouse Cy2 secondary antibody	#715-225-150	1:200	Jackson Immunoresearch
donkey anti-rabbit Cy3 secondary antibody	#711-165-152	1:200	Jackson Immunoresearch
donkey anti-chicken AF488 secondary antibody	#703-545-155	1:250	Jackson Immunoresearch
donkey anti-mouse AF488 secondary antibody	#A-21202	1:250	Thermofisher Scientific
donkey anti-rabbit AF568 secondary antibody	#A-10042	1:250	Thermofisher Scientific

Table S6. TaqMan assays used for qPCR.

Product	Reference	Company
TaqMan Gene Expression Assays (TaqMan Assay)	#4331182	Thermo Fisher Scientific
TaqMan Assay GAPDH	Assay ID: Hs99999905_m1	
TaqMan Assay NANOG	Assay ID: Hs02387400_g1	
TaqMan Assay POU5F1	Assay ID: Hs01654807_s1	
TaqMan Assay TUBB3	Assay ID: Hs00801390_s1	
TaqMan Assay SYP	Assay ID: Hs00300531_m1	
TaqMan Assay MAP2	Assay ID: Hs00258900_m1	
TaqMan Assay GFAP	Assay ID: Hs00909233_m1	
TaqMan Assay S100B	Assay ID: Hs00902901_m1	
TaqMan Assay ALDH1L1	Assay ID: Hs00201836_m1	
TaqMan Assay EXTL2	Assay ID: Hs00242124_m1	
